# Supplementary material for: Map of Enteropathogenic Escherichia coli Targets Mitochondria and Triggers DRP-1-Mediated Mitochondrial Fission and Cell Apoptosis in Bovine Mastitis
Source: Int J Mol Sci. 2022 Apr 28;23(9):4907. doi: 10.3390/ijms23094907 (PMC9105652; doi:10.3390/ijms23094907)
Supplement: Supplementary file 1 [file ijms-23-04907-s001.zip › ijms-1703493-supplementary.pdf]

Table S1. Major ***F***unction of ***E***ffector ***P***roteins**.**

Effector Proteins	Gene	Major Function Furing EPEC Infection
Tir	*tir*	Intimate adherence and actin pedestal formation
EspB	*espB*	Inhibition of myosin function, effacement of microvillus and anti-phagocytosis
EspF	*espF*	Membrane remodeling and actin nucleation
EspG	*espG*	Inhibition of recycling endosomes, protein secretion, and microtubule disruption
EspH	*espH*	Inhibition of opsono-phagocytosis and filopodial formation
EspZ	*espZ*	Inhibition of cell cytotoxicity
Map	*map*	Filopodia
